# Estimation of exposure to toxic releases using spatial interaction modeling

**DOI:** 10.1186/1476-072X-10-20

**Published:** 2011-03-21

**Authors:** Jamison F Conley

**Affiliations:** 1 Department of Geology and Geography, West Virginia University, 330 Brooks Hall, 98 Beechurst Ave., Morgantown, WV, USA, 26506

## Abstract

**Background:**

The United States Environmental Protection Agency's Toxic Release Inventory (TRI) data are frequently used to estimate a community's exposure to pollution. However, this estimation process often uses underdeveloped geographic theory. Spatial interaction modeling provides a more realistic approach to this estimation process. This paper uses four sets of data: lung cancer age-adjusted mortality rates from the years 1990 through 2006 inclusive from the National Cancer Institute's Surveillance Epidemiology and End Results (SEER) database, TRI releases of carcinogens from 1987 to 1996, covariates associated with lung cancer, and the EPA's Risk-Screening Environmental Indicators (RSEI) model.

**Results:**

The impact of the volume of carcinogenic TRI releases on each county's lung cancer mortality rates was calculated using six spatial interaction functions (containment, buffer, power decay, exponential decay, quadratic decay, and RSEI estimates) and evaluated with four multivariate regression methods (linear, generalized linear, spatial lag, and spatial error). Akaike Information Criterion values and *P *values of spatial interaction terms were computed. The impacts calculated from the interaction models were also mapped. Buffer and quadratic interaction functions had the lowest AIC values (22298 and 22525 respectively), although the gains from including the spatial interaction terms were diminished with spatial error and spatial lag regression.

**Conclusions:**

The use of different methods for estimating the spatial risk posed by pollution from TRI sites can give different results about the impact of those sites on health outcomes. The most reliable estimates did not always come from the most complex methods.

## Background

Environmental pollution data such as that collected by the United States Environmental Protection Agency's Toxic Release Inventory (TRI) have been used extensively for studies in environmental justice and medical geography [1]. These studies involved estimating an individual's or a community's exposure to pollution using the spatial information contained in the TRI database. Despite the use of this spatial information, the geographical theory used to guide the estimation of location-based exposure to pollution has frequently been limited to basic containment and buffer analysis, especially at the national scale. The aim of this research is to improve the spatial analysis of TRI data by incorporating distance decay effects derived from spatial interaction modeling in order to provide a more realistic approach to the estimation of location-based exposure to pollution, particularly airborne pollution. This is achieved by using several different functions for calculating this exposure and comparing the results when they are used in multivariate regression analyses with lung cancer mortality rates.

The different methods for estimating the risk at a location are evaluated because, while many studies have explored and demonstrated a link between environmental pollution and a variety of adverse societal and medical effects [1], understanding the nature of this relationship is equally important. As the variety of methods used to estimate these impacts attests, the nature of this relationship is not as well understood as the existence of the relationship. The form of this relationship greatly impacts the answers to questions that may arise from the discovery of a relationship, such as the extent to which rural counties experience adverse impacts from urban polluters. A visual cartographic comparison of some approaches has been explored by McMaster et al [2], although they do not make the statistical comparison carried out here.

### Prior work

Spatial analyses of toxic pollution data, whether for environmental justice or for medical geography, have typically used a simple spatial estimate of exposure. The exposure has been recorded as a binary variable (exposed or not exposed) either through spatial containment such that a person is exposed if they live in the same census tract or county as an industrial site [1,3-8], or a spatial buffer such that a person is exposed if they live within a threshold distance (e.g., 1 mile) of an industrial site [5,9-12]. Variations on the latter use multiple buffers to approximate decreasing risk with increased distance, or select a small number of neighborhoods at increasing distances which can be treated as samples from multiple buffers. This enables the study to reflect decreased exposure as the distance increases [1,9,13-15]. To provide a better measure of the impact of sites on a census tract, four studies [16-19] use a raster grid that can account for whether a site is in the center of the tract, or near an edge, and whether any sites are just over the border in neighboring tracts. These raster grids reflect the density of TRI sites around each raster cell, although the density is calculated using a small buffer, such as the density of sites within a one mile radius of the cell. A gradual decay of impact as distance increases is still lacking.

**Figure 1:**
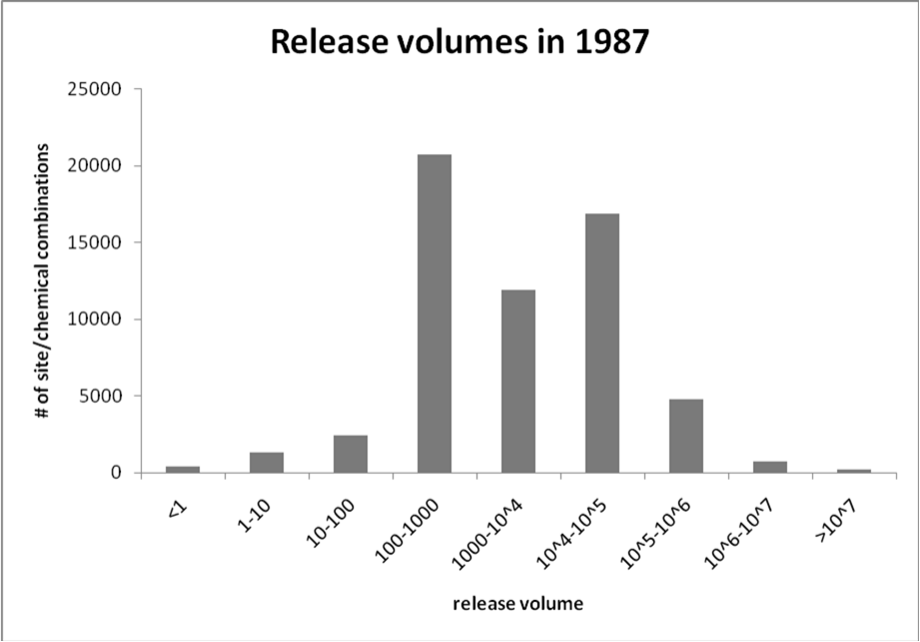
**Histogram of the volume of TRI releases for 1987**. Histogram of the TRI release volumes measured in pounds for 1987. Note the log scale on the horizontal axis.

Accounting for the volume of the release is another important factor missed by some TRI studies [6,10,16,18]. A binary approach that considers all TRI sites equally does not allow for gradients of risk, treats exposure to one site as equivalent to exposure to many sites, and does not account for the volume and toxicity of releases at each site. The release volumes vary by orders of magnitude (figure 1). Recognizing this, many researchers do account for varying release volumes from each site [7,9,12,20-27]. They often use variations of the spatial containment and buffer models described above which can incorporate the release volume (equations 1 and 2 respectively). Here, *k*_*ij *_is the impact of site *i *on county *j*, *t*_*i *_is the volume of releases at site *i*, *d*_*ij *_is the distance between site *i *and county *j*, and *T *is the threshold distance. As a result of this, most studies that use these techniques to account for the release volume still reflect a simple treatment of geography by not including distance decay effects. The toxic impacts of the different chemicals on human health vary as well [3,9,12,22-25,27], although this variation is not addressed in the current study.(1)(2)

To address these simplifying assumptions, Dent et al [26] have proposed using a GIS to combine atmospheric modeling with the release data and health outcomes and provide a detailed analysis of the potential effects and risks associated with TRI releases. Morello-Frosch et al [23,24] and Fisher et al [21] similarly incorporate atmospheric modeling in their analysis. These models are typically used for local, rather than national-scale analysis. The United States Environmental Protection Agency's Risk-Screening Environmental Indicators (RSEI) Model uses principles of atmospheric modeling to derive a level of risk across the entire United States [28]. This has been used by Abel [9] and Downey and Hawkins [27], and is used in this resesarch.

This background discussion is summarized by table 1, which shows that while distance decay approaches have been used, e.g. [20], containment and buffers are the most common with atmospheric modeling becoming more prevalent in local studies. Exponential and power-based distance decay approaches as found in spatial interaction modeling have, to the author's knowledge, not been used at all.

**Table 1:** Prior work summary

Reference	Volume?	Toxicity?	Function
[1]	N	Y^1^	Containment & multiple buffers

[3]	Y	Y	Containment

[4]	Y	Y^1^	Containment

[5]	N	Y^1^	Containment & buffer

[6]	Y^2^	N	Containment

[7]	Y	N	Containment

[8]	N	Y^1^	Containment & plume modeling ("census tract containing the site and its plume", p. 148)

[9]	Y	Y	Multiple buffers, RSEI

[10]	N	N	Multiple buffers

[11]	N	N	Containment & buffer (both distance boundary and areal apportionment)

[12]	Y	Y	Multiple buffers

[13]	n/a^3^	n/a	Neighborhoods of increasing distance

[14]	N	N	Multiple buffers

[16]	N	N	Distance-based raster

[17]	N	N	Multiple distance-based rasters & distance to nearest TRI facility

[18]	N	N	Multiple distance-based rasters

[19]	N	N	Distance-based raster

[20]	Y	Y	Cutter

[21]	Y	Y^1^	Atmospheric modeling

[22]	Y	Y	Containment

[23]	Y	Y	Atmospheric modeling

[24]	Y	Y	Atmospheric modeling

[25]	Y	Y	Atmospheric modeling

[26]	Y	N	Atmospheric modeling

[28]	Y	Y	RSEI

Papers summarized by the type of spatial interaction method applied and whether toxicity and the release volume are accounted for in the analysis.

^1^Analyzes one or more classes of chemicals separately

^2^Incorporates number of releases, but not volume

^3^Analyzes a single landfill rather than emission sites

### Spatial interaction modeling

In this research, I use a spatial interaction modeling approach that is more flexible than the binary approaches commonly used in spatial analysis of TRI data, yet is fast enough and generic enough to apply to the thousands or millions of release sites involved in a national scale study. Spatial interaction modeling was developed in economic geography to estimate the level of economic interaction between two towns [29-32]. The underlying assumptions are analogous to the physics theory of gravity. Just as two objects in space exert a stronger gravitational pull on each other as they increase in size and move closer to each other, two towns are expected to have a stronger level of economic interaction as the towns increase in size and as the distance between them decreases. These broad trends are applicable in many fields within geography, even though the specific functional form from physics (equation 3), may not be as useful as other functional forms.

These models are used to estimate the effect of each TRI site on each county. As the toxic release volume increases, the impact of that site on the county increases. Likewise, the impact of nearby sites is assumed to be greater than the impact of more distant sites, as from Tobler's First Law of Geography [33].

There are two common distance decay functions used to model spatial interaction, which control the rate at which the impact of a site decreases with distance. The first, taken from the physics model of gravity, is the power equation, in which the impact of a site is proportional to the size of the release and inversely proportional to the distance raised to a parameterized exponent (equation 3). Here, *α *and *θ *are positive constant parameters. The location of a county is given by its centroid. Because other functional forms may be more applicable than the gravitational form, exponential decay functions (equation 4) have also been developed and used.(3)(4)

The models in economic geography, such as those in Sen and Smith [31], give equations with a third term which in this work corresponds to the population of the county and a related positive constant β, such that the power model becomes  and the exponential model becomes *k*_*ij *_= *t*_*i*_^*α *^*p*^*β*^_*i *_exp(-*θ d*_*ij*_). Because I use age-adjusted rates of lung cancer rather than unadjusted counts for the dependent variable, these population terms are set to 1 and effectively removed from the equations.

The only application of a distance decay function to TRI data is a comparison of toxic releases and federally assisted housing which uses a quadratic distance decay function [20] (equation 5). This is referred to here as the Cutter function after the lead author of the publication in which it was first proposed. It uses a constant parameter, *θ*, controlling the rate of decrease, and a threshold distance beyond which the impact is zero. The equation given here modifies equation 1 from Cutter et al [18] to incorporate the volume of the release. As in the other equations, *k*_*ij *_is the impact of site *i *on county *j*, *t*_*i *_is the volume of releases at site *i*, *d*_*ij *_is the distance between site *i *and county *j*, and *T *is the threshold distance.(5)

**Figure 2:**
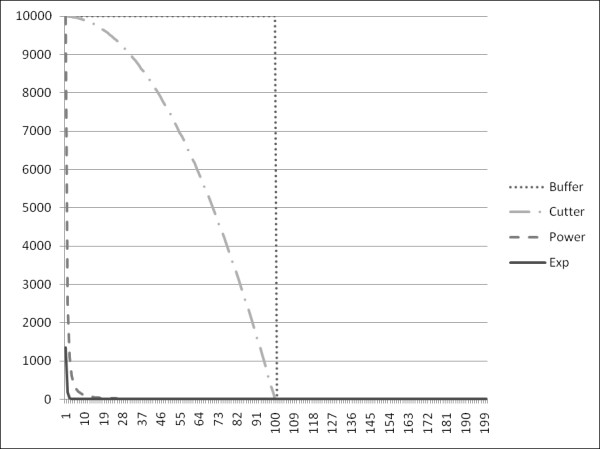
**Example graph of the distance decay functions**. Example graph of the four distance decay functions examined in this study: a buffer, Cutter's quadratic decay function, a power-based decay function, and an exponential decay function. All functions use 1.0 for α, 2.0 for θ, and 100 for *T*, with a release volume of 10,000.

Figure 2 shows the effect of increasing distance on all the models except the containment model. The parameters of the models shown are 1.0 for α, 2.0 for θ, and 100 for *T*, with a release volume of 10,000. More complex atmospheric models, which can incorporate distance decay concepts, have been used predominantly in studies at a local scale [21,23,24,26], with only the RSEI dataset used at the national scale [27].

## Methods

### Data used

Four sets of data are used in this paper. The first are lung cancer age-adjusted mortality rates from the National Cancer Institute's Surveillance Epidemiology and End Results (SEER) database [34]. These rates are from the years 1990 through 2006 inclusive. The second are TRI releases from 1987 to 1996. The years chosen for the TRI databases reflect a lag time between chronic exposure to toxic chemicals and the development of lung cancer. All data are temporally aggregated to the entire time series, rather than evaluating year-by-year temporal lags. The third are risk estimates computed by the EPA's RSEI program to be used as a basis for comparison against the spatial interaction estimates The RSEI data are the risk-related results calculated from airborne releases of chemicals that are flagged as carcinogenic and have a non-zero Inhalation Unit Risk.

The final dataset, the covariates, come from multiple sources. One source is the United States Census Area Resource File [35]. Thun et al [36] show variable risks for age, sex, and racial categories, so census data for the proportion of the population which is male and the proportion of the population which is non-white are included. Hendryx et al [37] note that lung cancer mortality is impacted by socioeconomic factors and access to health care, so additional covariates include the percent of the population with a less than high school education, the percent of the population with a college education, the percent of families below the poverty level, the unemployment rate, and the number of physicians per 1000 residents. Because smoking is the most significant risk factor for lung cancer [36], I also include the smoking rate of the county based on the BRFSS survey data from 2003 to 2006. Different regions of the United States have different rates of lung cancer [38], so the covariate data also includes spatial indicator variables recording whether a county is in the American south, northeast, Midwest, or western region, and whether a county is part of Appalachia, a regional designation from the Appalachian Regional commission which overlaps parts of the northeast, Midwest and southern regions. Due to a lack of data, information regarding personal movements is not included, although analysis comparing place of birth with place of death may partially account for this [39].

The Modifiable Areal Unit Problem [40] introduces difficulties into the interpretation at the county scale, especially in the larger counties in the western United States where the county centroid may be tens of miles away from the county's population center. Additionally, in these larger counties, the risk may vary within the county, and this variance is masked by calculating the risk at the county scale. However, some of the covariate data (e.g., the BRFSS-derived smoking rate) is not available at a finer scale, necessitating a county-level analysis. In the research presented here, the impacts of the Modifiable Areal Unit Problem and large county sizes are expected to have a similar impact across all models because all tests use the same spatial scale.

An examination using a synthetic dataset was considered, but the results of such a test would minimize the AIC in the situation reflecting the way the dataset is constructed (e.g., the impact falls off according to an exponential distance decay function), which may or may not correspond to a real world situation. Therefore, actual, rather than synthetic, data are used in this research.

### Methods Applied

Three sets of releases from 1987 to 1996 in the TRI database are used. The first is all releases flagged as carcinogenic. The second is all releases of chemicals identified as inducing lung cancer. These chemicals are those from a parallel study [41] plus beryllium and lead, which were identified as related to lung cancer by the lead author of [41] in a private communication. The total list of chemicals is arsenic, beryllium, 1,3-butadiene, cadmium, chromium, formaldehyde, lead and nickel. The third set of releases adds to the second set those releases identified as generic compound categories of elements in the first set. An example of this is a release of "arsenic compounds" in addition to releases of plain arsenic. The impacts of these three sets of releases on all counties in the contiguous United States were calculated using the containment, buffer, power, exponential and Cutter models given above. These release impacts are summed to create the cumulative impact on a county (equation 6), where *k*_*ij *_is the impact of site *i *on county *j *and *K*_*j *_is the cumulative impact on county *j*. Because the release amounts vary by several orders of magnitude and have an approximately lognormal distribution, as shown in figure 1, both the log_10_-transformed and the untransformed release volumes were tested.(6)

The calculated impacts and covariates are then used in multivariate regression models calculated with the R software package [42]: ordinary least squares (OLS) linear regression, general linear model regression (GLM), spatial lag regression, and spatial error regression. The latter two incorporate spatial dependence in the regression model and are detailed below. There is spatial autocorrelation in the response variable (Moran's I = 0.69, p < 0.01), demonstrating the existence of spatial dependence and suggesting the applicability of the spatial regression techniques. Geographically Weighted Regression [40], which can vary the regression coefficients across the study area was not applied because it is unlikely that the nature of the relationship between toxic releases and mortality changes across the country.

This situation is not strictly one of evaluating a single function against a null hypothesis of zero impact from toxic emissions, but is rather evaluating many different functions against both each other and the null hypothesis. This makes the task more akin to model parameter optimization than traditional statistical hypothesis testing. It is considered here that, of the different functions and their parameterizations, the most appropriate representation is the one that minimizes the Akaike Information Criterion (AIC) of a regression test in which the modeled risk is one of the independent variables. In all the regression tests used in this comparison, the remainder of the independent variables are the demographic, behavioral and regional covariates, and the dependent variable is lung cancer mortality.

Experiments testing many parameterizations of the buffer, Cutter, power, and exponential functions were used to guide the results given here. These parameterizations are for the contiguous United States, and were evaluated on which parameterizations gave the lowest AIC values when combined with the covariate data in an OLS regression using the lung cancer mortality rate as the dependent variable. The same tests are conducted for the containment and RSEI approaches to risk estimation for comparison. AIC values were also computed for generalized linear model regressions, although none of the generalized linear model regressions produced lower AIC values than linear regression. As a result, the generalized linear model regressions are not further discussed. Preliminary experiments (not presented) demonstrated that the parameterizations that perform well for OLS regression also typically perform well for the spatial lag and spatial error regressions. The tests presented here evaluate *α *and *θ *values of 1.0, 1.5, 2.0, 2.5, 3.0, 3.5, 4.0, 4.5, and 5.0, and distance thresholds of 5, 10, 15, ... 500 miles (8, 16, 24, ... 805 km) for the buffer and Cutter functions. After the parameterizations were found, spatial lag regression and spatial error regression models were computed. Also, linear regression models for each of the rural-urban continuum codes from the United States Department of Agriculture. Lastly, maps of proportion of the TRI impact for each county that originated in release sites located in urban areas were produced. This allows an examination of whether the impact of sites in urban areas is limited to those cities, or whether it extends far into the surrounding rural areas, and demonstrates that some environmental justice questions are not robust to the choice of risk model.

### Spatial Regression Methods

The two spatial regression methods, spatial lag and spatial error regression, both account for the spatial autocorrelation that is almost always present in geographic data by adding a term to the regression equation. This spatial autocorrelation can be the result of diffusion effects of the dependent variable, which is unlikely in this situation, or the result of risk factors which have not been accounted for elsewhere in the regression model inducing spatial autocorrelation of the dependent variable [43]. The standard OLS regression (equation 7) estimates the dependent variable, which is the lung cancer mortality rate, as a linear combination of the independent variables, here the TRI interaction term and the demographic covariates. The county is *j*, the dependent variable at county *j *is *Y*_*j*_, the independent variables at county *j *are *X*_*j,a*_, *ε*_*j *_is the error term, and *β*_*0 *_... *β*_*n *_are the regression coefficients.(7)

Spatial lag regression incorporates the autocorrelation directly into the model by including a term where the dependent variable at county *j *is dependent not only on the independent variables at county *j*, but also on the dependent variable values of county *j*'s neighbors [44]. The neighbors are defined by a weights matrix typically using one of the following three options: (1) all counties which share a border with county *j *as its neighbors, (2) all counties within a threshold distance of county *j *as its neighbors, or (3) the nearest neighbors of county *j*. Here, option (2) is used a distance threshold of 92 miles (148 km), which is the minimum distance that ensures all counties have at least one neighbor. Option (1) is also used considering shared corners (eg, the Four Corners meeting between Arizona, New Mexico, Colorado and Utah) as neighbors, which is called "queen contiguity" to assess the sensitivity of the results to the choice of weights matrix. Thus spatial lag regression uses the dependent variable values of the neighbor counties to calculate a new independent variable for county *j*, giving equation 8, where *ρ *is a coefficient describing the strength of the spatial autocorrelation, *w*_*j,k *_is the spatial weight between counties *j *and *k *(typically 1 for neighbors and 0 for non-neighbors, but a distance decay form for the weights is possible), and *N*_*j *_is the neighborhood of counties around county *j*. The *ρ *coefficient can be estimated in the same way that the *β *coefficients are estimated. A computationally efficient approach is given in Smirnov and Anselin [45].(8)

Spatial error regression (equation 9) works similarly to spatial lag regression, except the autocorrelation term applies to the error terms of the neighboring counties rather than their dependent variable values [46]. Because of the circular dependence of the error terms (ie, if county *j *is in county *k*'s neighborhood and vice versa, the value of *ε*_*j *_is affected by the value of *ε*_*k *_while the value of *ε*_*k *_is affected by the value of *ε*_*j*_), standard estimation techniques will not work. An estimation procedure for this is also given by Smirnov and Anselin [45].(9)

## Results

Table 2 presents the parameterizations that gave the lowest AIC values and thus are used for further analysis. The results shown use the lung carcinogens with compounds dataset. All three TRI release sets described above (all carcinogens, lung carcinogens, lung carcinogens with compounds) were evaluated as were the log_10 _transformed release values, and the lung carcinogens and related compounds gave the lowest overall AIC values. While the containment approach was best fit with the log-transformed releases of lung carcinogens and related compounds, and the exponential and power functions had the best fits with releases of all carcinogens, the improvements were minimal; the differences in AIC are less than 2.0 for containment and the exponential function and approximately 10.0 for the power function. Therefore, to ensure consistency in the later tables, the untransformed releases of lung compounds with carcinogens are used.

**Table 2:** Model parameterizations

	buffer	power	exponential	Cutter
α		1	1	1

θ		1	1	5

T	500 miles (804 km)			500 miles (804 km)

Parameterizations of the models which are the best performing for each distance decay function, and are thus used in the analysis.

Table 3 shows the R-squared values, the Akaike Information Criterion, and the probabilities that the spatial interaction terms are non-zero. For each regression model (OLS, spatial lag, or spatial error), the best-performing distance decay function is highlighted in bold. In all cases, this was the buffer model. Table S1 in Additional file 1 shows the equivalent table for the queen contiguity weights matrix. The choice of weights matrix did not alter the results for most decay functions, only substantially increasing the AIC of the buffer model, but not enough to make another decay function better. As such, the change in weights matrix did not alter conclusions about which decay function performed best. Table 4 gives the full regression results for the overall best parameterization: the buffer model at 500 miles (804 km). This table also gives values of each independent variable's variance inflation factor (VIF). Since all the VIFs are less than 10, collinearity is not problematic in this model. As some of the covariates did not have significant coefficients in the best-performing model, the least significant covariate was iteratively removed from the model until all independent variables were significant, producing the model in the right side of table 4. Similarly, non-linear functions of each of the independent variables were also applied to each of the six parameterizations in Table 3, following [47]. While the fits are improved (minimized AIC = 22126.54 with the buffer function), the more complex regression models do not alter the conclusions about which spatial interaction models perform well and which perform poorly. Table S2 in Additional file 2 gives the best performing model results. Table 5 gives the R-squared values of the OLS regressions by urban-rural code. The values of these codes are in table 6.

**Table 3:** Regression results

OLS	no term	**contain**.	buffer	power	**exp**.	Cutter	RSEI
R-squared	0.4596	0.46	**0.5224**	0.4623	0.4596	0.5178	0.4599

Akaike Info. Criterion	22873.61	22873.22	**22498.13**	22860.47	22875.59	22527.45	22874.03

probability of TRI term	n/a	0.122677	**<.000001**	0.000104	0.877151	<.000001	0.209438

Spatial Lag							

Akaike Info. Criterion	22875.6	22875.21	**22350.12**	22852.86	22876.37	22529.4	22876.02

probability of TRI term	n/a	0.91113	**<.000001**	0.96198	0.91903	0.83221	0.9158

Spatial Error							
Akaike Info. Criterion	22873.95	22873.51	**22298.14**	22850.64	22874.72	22525.14	22874.57

probability of TRI term	n/a	0.19085	**<.000001**	0.13587	0.19759	0.038097	0.22659

Results for the multivariate ordinary least squares, spatial lag, and spatial error regressions of age-adjusted lung cancer mortality versus covariates and risk estimates from releases of lung carcinogens and related compounds calculated with each spatial interaction model. Bold entries indicate which spatial interaction model performed best. Note that lower values for the Akaike Information Criterion are preferred.

**Table 4:** OLS Regression results

	VIF	**Coeff**.	Std. Error	t value	Pr(>|t|)		**Coeff**.	Std. Error	t value	Pr(>|t|)	
Intercept		33.53	5.823	5.758	9.39E-09	***	35.96	1.393	25.808	< 2E -16	***

% no high sch.	6.165	-0.007	0.0416	-0.171	0.8646						

% in poverty	4.306	-0.295	0.0517	-5.706	1.27E-08	***	-0.308	0.038	-8.054	1.13E-15	***

% unemployed	2.082	1.386	0.0818	16.948	< 2E -16	***	1.365	0.079	17.315	< 2E -16	***

% non-white	1.867	-0.022	0.0157	-1.384	0.1665						

Appalachian	1.613	-6.539	0.6447	-10.14	< 2E -16	***	-6.318	0.591	-10.684	< 2E -16	***

College educ.	3.401	-0.396	0.0494	-8.012	1.59E-15	***	-0.396	0.039	-10.089	< 2E -16	***

Smoking rate	1.708	0.526	0.052	10.116	< 2E -16	***	0.540	0.051	10.578	< 2E -16	***

South	5.119	0.822	0.7923	1.037	0.2996						

Midwest	3.821	-6.51	0.7893	-8.247	2.39E-16	***	-6.894	0.484	-14.234	< 2E -16	***

West	4.197	-1.824	0.8067	-2.261	0.0238	*	-2.219	0.613	-3.621	0.0003	***

Physicians/1000	1.675	1.518	0.2203	6.891	6.71E-12	***	1.461	0.217	6.727	2.06E-11	***

% male	1.115	0.0451	0.1122	0.402	0.6877						

Risk estimate	3.772	2.9E-07	1.5E-08	20.208	< 2E -16	***	2.9E-07	1.3E-08	22.525	< 2E -16	***

Results for the multivariate ordinary least squares regression of age-adjusted lung cancer mortality versus covariates and risk estimates from lung carcinogens and related compounds calculated with the buffer model. This is shown as it minimizes the AIC across all decay functions (Table 3).

Significance codes: 0.0001 '***' 0.001 '**' 0.01 '*'

n = 3057

**Table 5:** OLS regression R-squared values by rural-urban code

Code	no term	**contain**.	buffer	Cutter	**exp**.	power
0	0.4971	0.4971	**0.5682**	0.5493	0.5100	0.4978

1	0.4113	0.4318	0.4795	**0.4840**	0.4119	0.4345

2	0.4654	0.4655	**0.5143**	0.5065	0.4655	0.4690

3	0.4618	0.4678	**0.5216**	0.5089	0.4621	0.4660

4	0.4815	0.4818	**0.5514**	0.5210	0.4828	0.4841

5	0.5886	0.5905	**0.6753**	0.6425	0.5899	0.5939

6	0.3990	0.3992	**0.4562**	0.4436	0.3997	0.3990

7	0.5231	0.5231	**0.5882**	0.5862	0.5234	0.5232

8	0.5555	0.5628	**0.5829**	0.5749	0.5560	0.5578

9	0.5741	0.5746	0.5892	**0.5909**	0.5748	0.5764

Results for multivariate ordinary least squares regression of age-adjusted lung cancer mortality versus covariates and risk estimates from releases of lung carcinogens and related compounds, separated by the county's 1993 rural-urban continuum code (see Table 6). Bold entries indicate which spatial interaction model performed best.

**Table 6:** Definition of each rural-urban code

Code	Description
	Counties in metropolitan areas

0	Central counties of metropolitan areas of 1 million population or more.

1	Fringe counties of metropolitan areas of 1 million population or more.

2	Counties in metropolitan areas of 250,000 to 1 million population.

3	Counties in metropolitan areas of fewer than 250,000 population.

	Counties not in metropolitan areas

4	Urban population of 20,000 or more, adjacent to a metropolitan area.

5	Urban population of 20,000 or more, not adjacent to a metropolitan area.

6	Urban population of 2,500 to 19,999, adjacent to a metropolitan area.

7	Urban population of 2,500 to 19,999, not adjacent to a metropolitan area.

8	Completely rural or less than 2,500 urban population, adjacent to a metropolitan area.

9	Completely rural or less than 2,500 urban population, not adjacent to a metropolitan area.

The interpretation of each 1993 rural-urban continuum code used in Table 5.

**Figure 3:**
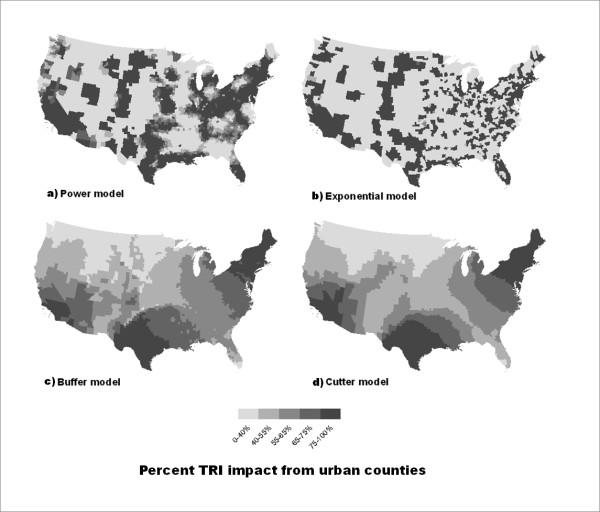
**Percent of TRI impact from urban releases**. Percent of TRI impact from urban releases using the different decay functions. Darker counties have a higher percentage of their impact coming from release sites in urban counties, while lighter counties have a higher percentage of their impact coming from release sites in suburban and rural counties.

Maps of the percent of TRI impact in each county that is due to source locations in urban counties according to each of the distance decay functions are given in figure 3. The buffer, Cutter, exponential and power functions using the parameterizations in table 2 are shown. The darker counties have a greater percent of their impact from releases in urban counties, whether the total impact is high or low.

## Discussion

The buffer and Cutter functions outperform the containment, power and exponential functions (Table 3). This improvement is notable both for the R-squared values of the OLS regressions and the Akaike Information Criterion (AIC) for all regressions. Because the AIC penalizes regression models with more parameters, lower values are preferred. These functions also outperformed the RSEI risk-related results values, which was not the expected outcome. Other RSEI products, the hazard, modeled hazard, and modeled hazard*population product were also tested, but all performed worse than the buffer and Cutter functions. However, the power and exponential functions from the spatial interaction literature did not perform much better than leaving out the toxicity term, and occasionally even increased the AIC value, which may result from the coarse resolution of the county-level dataset. These two functions may yet be useful at a finer scale.

The improvement of the buffer and Cutter functions over the RSEI data demonstrates that despite the difficulties posed by the Modifiable Areal Unit Problem and the size of large western counties obscuring variation of risk within the county, these spatial interaction approaches may still be an accurate reflection of the risks posed by TRI facilities. It should be cautioned that while this work demonstrates a relationship between TRI facilities and lung cancer, it does not yet indicate a causal link, nor does it indicate that the best-fitting risk estimation method, a large buffer around the TRI site, has the strongest causal relationship with lung cancer mortality.

Additionally, AIC values are better for the spatial regression techniques compared to the OLS regression values. However, including the TRI term in the spatial regression techniques does not lead to as much improvement over the base case of no interaction term. Even so, the improvement in the buffer model gives the spatial error regression of the buffer model the lowest AIC value. Both the Moran's I spatial autocorrelation statistic given above and the lower AIC values for the spatial regression techniques indicate that there is spatial dependence in lung cancer mortality. Moreover, this spatial dependence is not accounted for by the independent variables. This dependence is most likely the result of one or more additional spatial processes affecting lung cancer that are not accounted for in these data, rather than a simple diffusion or contagion process of lung cancer itself. The limited improvement from adding the TRI impacts strengthens the suggestion that there remain geographic processes affecting lung cancer that are not accounted for in these datasets. While it is not executed in this study, GWR may also reveal further evidence of confounding processes by revealing interactions with modeled covariates via non-stationary regression coefficients.

As with the different regression methods, the buffer and Cutter functions have the best R-squared values across the entire range of rural-urban continuum codes (Table 5). Also, category 5, defined as counties containing a larger town (more than 20,000 residents) but which are not adjacent to a metropolitan area, has much higher R-squared values than the other rural-urban codes across all models. It is not yet clear why this would be the case.

These results suggest that changing the method used to estimate risk will change the representation of the spatial impacts of the TRI sites on public health. As others have noted, the scope and scale of analysis can substantially impact the results [48], so researchers should be cautious when generalizing these findings at a county scale and national scope to more local scales and scopes. Nonetheless, researchers using the TRI dataset to estimate the health risks from pollution should carefully consider the method used to estimate the risk, as the most sophisticated model used here, the RSEI data, did not provide the lowest AIC values.

The maps in figure 3 display the percent of the TRI impact on each county from sources in urban areas calculated using the functions that performed best in the earlier results. As estimated by these models, the potential effects of pollution from urban TRI releases extend far beyond the limits of the urban areas. However, the extent varies depending on which function is used and how it is parameterized, highlighting the importance of using an appropriate function. In the power and exponential maps (figure 3a and 3b), the impacts from urban release sites are more limited to urban areas and the nearby rural communities. In both the buffer and Cutter maps (figure 3c and 3d), rural areas in the northeastern and southwestern United States, have between 75 and 100% of their estimated TRI impact from release sites in urban areas. These extended effects of urban areas are related to the large radii used in the distance decay functions. Additional work is needed to examine the environmental toxicology to determine whether the chemicals being released could travel such large distances or whether these models are simply capturing spatial dependence of the outcome that is induced by a confounding spatial process.

Future work will investigate the parameterization choices of the functions. This ad hoc approach to parameterization-examining different possibilities of the α, θ and threshold parameters-is not the ideal approach. Statistical approaches to finding the optimal α and θ parameters can be incorporated to improve the spatial interaction models that are generated [29,30,32]. A geostatistical approach can be applied to determine the decay function form and parameters. A correlogram plot comparing the distance between two counties and the difference between their mortality rates or their residuals from a regression function could be used to parameterize the function. Additionally, subsets of the correlogram could be examined separately to investigate anisotropy and non-stationarity. However, with both the ad hoc approach in this paper and a statistical model-fitting approach, using the data to optimize the parameters and then using those parameters to analyze the same data introduces circularity into the model-fitting process that would best be avoided.

A more theoretically sound approach would be to vary the α and θ parameters based on the properties of the toxic chemicals that are released. Varying α is similar to methods used somewhat frequently to account for the different toxicity of the chemicals released [2,8,11,20-22], although the studies cited here use multiplicative rather than exponential modifiers (*α t*_*i *_instead of *t*_*i*_^*α*^). In each case, higher values of *α *correspond to more toxic chemicals. Different studies have made this adjustment using different references, including American Conference of Governmental Industrial Hygienists Threshold Limit Values [3,22], a chronic toxicity index [12], an inhalation unit risk [23], a lifetime cancer risk [24], and the RSEI model [9,27]. Similarly, θ and *T *can be varied to reflect differences in airborne transport of the chemicals. If a chemical travels more easily and farther, lower values of θ and higher values of *T *can be used. These parameters can also be varied based on the direction from the release site to the affected community, thus incorporating anisotropy.

Ongoing work includes the refinement of at-risk population estimates using the LandScan USA population dataset [49] which can explore variation missed by county-level populations unable to capture fine-scale risks. For example, if a chemical is only present in the atmosphere within a mile of the release site, any county-by-county analysis will be problematic because the spatial resolution of county-level data are coarser than a square mile. The LandScan dataset provides population estimates at a 3 arc-second resolution (roughly 90 meters). This can then provide improved estimates of the number of people within one mile of the release site instead of assigning the impact of a release site on the county as if everybody lived at the centroid of the county. This approach will have stronger effects on the power and exponential models because they have more rapid decreases in the impact as one travels farther from the release site (figure 2). This ongoing work also incorporates the adjustments given above varying the parameters to account for properties of the chemicals released and local climatic conditions to account for prevailing wind directions.

## Conclusions

The research in this paper demonstrates that the use of simple containment techniques for estimating the spatial risk posed by pollution from TRI sites as well as the RSEI risk-related results can give misleading results about the impact of those sites on health outcomes. This is done through a comparison of multivariate regression results using inputs of six different functions for estimating the impact of a release site on a county: containment, buffering, the quadratic distance decay function proposed by Cutter et al [20], an inverse power distance decay function, an exponential distance decay function, and the RSEI risk-related results. The buffer and Cutter approaches consistently performed the best among these methods. The effects of this function choice are also demonstrated through mapping the percent of the overall impact that comes from urban TRI sites for all models except containment. As refinements to the parameterization process are made, the utility of more theoretically sound spatial interaction models will improve further.

## Competing interests

The author declares that he has no competing interests.

## Supplementary Material

Additional file 1 **Table S1 - Results for the multivariate ordinary least squares, spatial lag, and spatial error regressions of age-adjusted lung cancer mortality versus covariates and risk estimates from releases of lung carcinogens and related compounds calculated with each spatial interaction model**. Spatial regressions here use queen contiguity matrix to determine whether two counties are neighbors. Bold entries indicate which spatial interaction model performed best. Note that lower values for the Akaike Information Criterion are preferred. Click here for file

Additional file 2 **Table S2 - Results for the multivariate ordinary least squares regression of age-adjusted lung cancer mortality versus nonlinear functions of both covariates and risk estimates from lung carcinogens and related compounds calculated with the buffer model**. This is shown as it minimizes the AIC across all decay functions (Table 3). This is equivalent to Table 4 in the main document, but includes natural logarithms (e.g., log(pov)), squared values (e.g., pov2) and cubed values (e.g., pov3). Click here for file
